# When Mind and Body Speaks: Understanding Dissociative Neurological Symptom Disorder-ICD–11

**DOI:** 10.1192/bjo.2025.10709

**Published:** 2025-06-20

**Authors:** Sumira Qambar Bokhari, Aysha Butt, Qambar Murtaza Bokhari, Rehma Shanze Alam, Aimen Saleem

**Affiliations:** 1 Services Institute of Medical Sciences, Lahore, Pakistan.; 2 Omar Hospital, Lahore, Pakistan; 3 Mughal Eye Hospital, Lahore, Pakistan; 4 Services Hospital, Lahore, Pakistan

## Abstract

**Aims:** Dissociative Neurological Symptom Disorder (DNSD), also known as Conversion Disorder, is a common diagnosis among mental health patients in Pakistan. Despite its prevalence, research on DNSD, especially regarding patient experiences, is limited. Family-related stressors are significant contributing factors in its development, with familial discord playing a key role in triggering symptoms.

**Methods:** A 24-year-old female was referred by a neurologist after presenting to the outpatient department in a wheelchair due to a fear of falling. She reported symptoms including jerky body movements, episodes of apparent loss of consciousness, diarrhoea, weakness, visual disturbances, headaches, and palpitations, which had persisted for over two years. Despite multiple consultations, no organic cause was identified. She had been prescribed various medications with no improvement. Upon evaluation, a diagnosis of DNSD was made, compounded by significant emotional stress, particularly familial discord. A multidisciplinary approach, involving specialists in neurology, ophthalmology, ENT, and gastroenterology, helped rule out underlying physical conditions. Her pre-morbid history indicated high academic achievement but chronic familial stress.

**Results:** This case emphasizes the importance of recognizing psychosomatic presentations in patients with unexplained neurological and physical symptoms. Despite extensive negative investigations, the patient’s psychological stressors, such as familial discord and fear of stigma, were key contributors to the onset of her symptoms. The patient’s fear of walking further exacerbated her physical disability. Treatment included sertraline, mirtazapine, and short-term benzodiazepines for anxiety and depression. Psychological therapies such as Cognitive Behavioural Therapy (CBT), deep breathing, safe-place visualization, imaginal exposure, systematic desensitization, and gratitude journalling were used to address emotional issues and reduce disability. This comprehensive approach resulted in symptom resolution, and the patient was symptom-free within 18 months, now pursuing an MPhil degree.

**Conclusion:** This case highlights the value of a multidisciplinary approach in diagnosing and treating complex physical symptoms with unclear aetiology. Addressing underlying psychological stressors and utilizing appropriate psychosocial interventions significantly improved the patient’s condition. Early identification and holistic management combining medical, psychological, and emotional support are crucial for effective treatment of DNSD.

